# Determination of Plate Corrosion Dimension Using Nd:YAG Pulsed Laser-generated Wavefield and Experimental Dispersion Curves

**DOI:** 10.3390/ma13061436

**Published:** 2020-03-21

**Authors:** Kassahun Demissie Tola, Dai Quoc Tran, Byoungjoon Yu, Seunghee Park

**Affiliations:** 1 School of Civil, Architectural Engineering and Landscape Architecture, Sungkyunkwan University, Suwon 16419, Korea; kastolla@skku.edu (K.D.T.); daitran@skku.edu (D.Q.T.); 2 Department of Convergence Engineering for Future City, Sungkyunkwan University, Suwon 16419, Korea; mysinmu123@skku.edu

**Keywords:** corrosion damage, ultrasonic wave propagation imaging, root mean square, dispersion curves, Rayleigh–Lamb equation, Fourier transform

## Abstract

Corrosion detection using a pulsed laser scanning system can be performed via ultrasonic wave propagation imaging. This method outputs illustrations of the wave field within the host structure; thus, it can depict wave–corrosion area interactions. Additionally, post-processing can be performed to enhance the visualization of corroded areas. The wavefield energy computed using RMS (Root Mean Square) is a validated post-processing tool capable of displaying the location and area of corrosion-damaged regions. Nonetheless, to characterize corrosion, it is necessary to determine its depth. The measurement of depth in conjunction with that of the corroded area via the RMS distribution enables the determination of all dimensions of corrosion damage. Thereafter, the flaw severity can be evaluated. This study employed a wavefield within a plate on which corrosion was developed artificially to generate frequency–wavenumber dispersion curves. The curves were compared with their counterparts from a corrosion-free plate. Alternatively, they could be compared with dispersion curves drawn using the depth and material properties of a pristine plate via a computer program. Frequency–wavenumber pairs were extracted from the dispersion curves produced using the portion of the wavefield within the corroded area. These were inserted into the Rayleigh–Lamb equation, from which depths were calculated and averaged.

## 1. Introduction

In an ultrasonic wave-based NDE (Non Destructive Evaluation), damages on plates and plate-like structural components are assessed primarily using ultrasonic guided waves rather than other types of ultrasonic waves. Lamb waves are generally known as plate waves because they exist in a thin plate-like isotropic material and are guided by free upper and lower surfaces. In addition, such waves are capable of traversing a relatively large distance, compared to other types of waves, before attenuation [1,2,3]. Lamb waves are sensitive to both surface and subsurface defects of a plate, which also makes them preferable [4].

The pulsed laser generation of Lamb waves is an extensively applied excitation technique for the damage inspection of plates. The structural response of a plate can be measured either through a laser Doppler vibrometer [5] or a PZT (Lead Zirconate Titanate) sensor [6]. These actuation-sensing pairs can be employed to scan a predefined area of a specimen by introducing a galvanometer into the configuration. A galvanometer equipped with a tilting mirror is effective for 2D scanning. There are four schemes of scanning as described in [5]: fixed-point PZT excitation and scanning laser sensing, scanning laser excitation and fixed-point PZT sensing, fixed-point laser excitation and scanning laser sensing, and scanning laser excitation and fixed-point laser sensing. In general, these scanning techniques are aimed at obtaining wavefield information regarding the specimen from the generated ultrasonic wave. Once the wavefield information is recorded, signal processing methods are adopted to extract the feature that best describes the ultrasonic wave–defect interaction so that the damage can be detected and quantified.

One of the aforementioned signal processing methods is UWPI (Ultrasonic Wave Propagation Imaging). The aim of this method is to acquire complete waveform data over a region of interest and display the acquired waveform data using images [7]. Wavefield images represent instantaneous waveforms in the regions captured by the images. In addition, a wave propagation movie can be produced by displaying the images in rapid succession [8]. Thereby, UWPI reveals the wave–flaw interaction. It is therefore considered as a tool for damage detection. In addition, the dimensions of the damaged area can be partially determined from the images. However, additional post-processing is required for characterizing the structural damage and assessing the severity level. The information obtained from such processing is highly essential for predicting the service life of a structural element.

In the scenario explained above, corrosion damage can be detected and visualized using a pulsed laser scanning method and a flaw imaging technique. Early works detected corrosion damage by using fixed point excitation and a stationary probe for sensing. Images are scanned and reconstructed by repeatedly measuring a structural response to the generated wave by moving the sensor successively inside the scanning area. For example, Silva et al. detected hidden corrosion in an aircraft aluminum plate structure by applying a short laser pulse focused on a line spot as a source of ultrasonic guided waves; they employed the heterodyne Mach–Zehnder interferometer for measuring the out-of-plane surface displacements [9]. Subsequently, arrays of sensors were utilized as both the excitation and sensing components. Image reconstruction is feasible because the array distribution is such that a spatial sampling will not suffer from aliasing [10,11,12]. The shift to this technique is because a sensor acquires signals locally, irrespective of its type. Moreover, it generally tends to provide inadequate information for assessing structural damage and, therefore, the identification results obtained via such sensors are prone to errors [13]. Subsequently, the scanning schemes mentioned in the preceding paragraphs, which are also described in [5], became extensively used [14,15,16]. The application of laser ultrasonic-based scanning enabled the production of ultrasonic wavefield images with high spatial resolutions. In addition, damage diagnosis could be performed without baseline data from the intact condition [5]. Furthermore, the feasibility of flaw detection of complex structures as well as flaw detection in inhospitable environments was enhanced [17].

Damage detection, localization, and visualization are the major merits of the pulsed laser scanning system. Post-processing should be proposed and performed for further damage characterization and size quantification. In the case of corrosion, size quantification refers to the determination of depth because it is feasible to measure the areal extent of the corrosion via the flaw imaging step. That is, it refers to the determination of the thickness of the specimen at the location where corrosion has occurred. Thickness determination using Lamb waves was the subject of investigation in a number of previous works. In their study in the early 1990s, Dewhurst et al. demonstrated the estimation of the thickness of a thin metal using a laser-generated ultrasound [18]. Gao et al. demonstrated a 2D Fourier transform method for producing a Lamb wave dispersion curve. They determined the thickness of a thin plate using this curve [19]. In their research, a method for approximating the dispersion relations for the $S_{0}$ and $A_{0}$ modes, from a previous work by Hutchins et al., was employed for computing thickness [20]. Deán et al. determined the thickness of an aluminum plate by fitting experimental data to the theoretical dispersion curves of a Lamb wave. They applied the wedge method of wave generation and a pulsed laser electronic speckle pattern interferometry system to map the displacement field on the plate surface [21]. In particular, corrosion depth estimation was performed by applying various algorithms for determining the thickness of the specimen at the region where corrosion had occurred. Zhu et al. proposed a quantitative evaluation of thinning in aluminum caused by corrosion by using the plate frequency compensation concept [22]. A few previous works have performed corrosion depth estimation by using a pulsed laser for wave generation. Liu et al. demonstrated the determination of the thickness of the internal corrosion layer of a Ni superalloy material by considering a high-frequency ultrasonic wave using laser ultrasonic and piezoelectric pulse-echo [23]. The study compared the performance of the two aforementioned methods and concluded that the laser ultrasonic method exhibits a higher detection resolution.

Converting wavevield time histories to their corresponding frequency-wavenumber maps through Fourier transform is one of the approaches to obtain the dispersion curves. Alleyne and Cawley showed the method of extracting wavenumber dispersion using Fourier transformation of the time history of the waves received at a series of equally spaced positions along the propagation path [24]. The method had since then become popular and applied by many studies. Hora et al. reported the method for determination of Lamb wave dispersion curves by using Fourier transform [25]. The extraction of dispersion curves from an ultrasonic wavefield generated using laser was applied by Flynn et al. for characterizing structural defects [26]. The study demonstrated the possibility of generating the estimates of the local dispersion curves. Delamination of laminated composites was also detected using guided wave dispersion extraction by using scanning laser Doppler vibrometer (SLDV) [27].

This study focused on corrosion damage sizing, which is based on UWPI, to determine area information and experimental dispersion curves for obtaining depth. In principle, this study was a continuation and upgrade of a study previously performed and published by our research team [28]. The main objective was to use the damage-related information acquired by applying UWPI, i.e., detection and localization, as an input in the post-processing stage. An Nd:YAG pulsed laser was used to scan a region on the specimen. An acoustic emission sensor was used to measure the ultrasonic wave signal. The UWPI method demonstrating the propagation of a wave radially outward from the location of the sensor was employed with the assumption that the elastodynamic reciprocity theorem holds. The weighted root mean square (RMS) of the signal was computed for each position on the laser scanning path. The (x,y,RMS) plot clearly indicated the location and areal extent of the corrosion damages. The UWPI and RMS results were used to visualize the pattern of the propagation of the wave through the corroded area and extract the boundaries of the areas, respectively. Using these, the features of wave propagation inside each corroded region were extracted, and further analysis was carried out to relate the wave information with the thickness of the area. In this study, further analysis refers to the preparation of an experimental dispersion curve for a wave that is only within the damaged area.

## 2. Principle of Ultrasonic Wave Propagation Imaging

**Figure 1:**
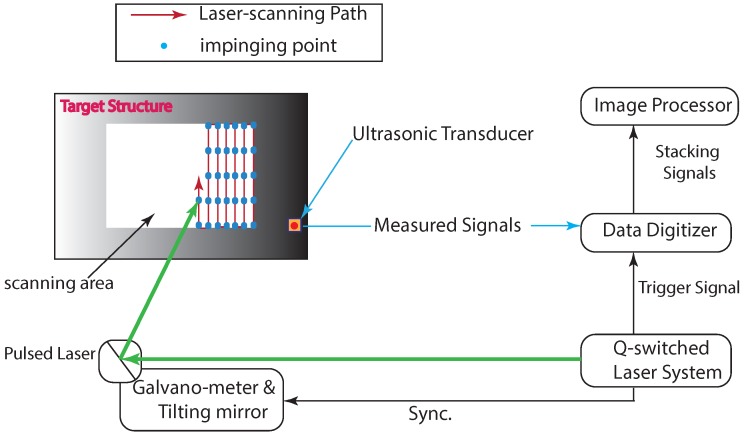
Laser-induced UWPI system.

In the scanning approach of wavefield data acquisition, the ultrasonic wave generation and propagation that results from the impingement of an Nd:YAG pulsed laser on a target surface can be configured to repeat at all the spatial points along a scanning path. Furthermore, a sensor measures the amplitudes of the waves reaching it from all the locations where waves are generated and stores them as separate waveforms. In general, the system comprises the components that are illustrated in Figure 1. The role of the galvanometer and tilting mirror is to enable the scanning of a 2D area. This is accomplished by rotating the mirror about the horizontal and vertical axes. The scanning area information is fed and controlled from a computer in which a GUI (Graphic User Interface) is setup for managing the system.

**Figure 2:**
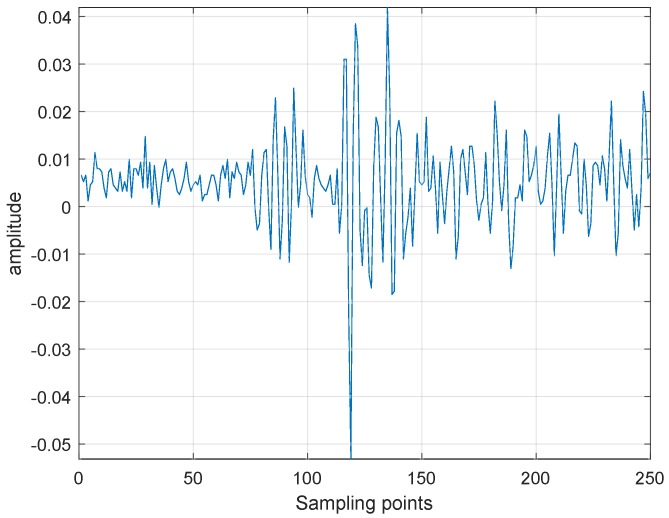
Laser generated waveform corresponding to a single laser impingement point.

The measured signal representing the wave generated at each point on which the laser impinges is stored in a manner such that each of the stored data points contains the temporal information of the wave as it travels from the source to the sensor. As an illustration, the waveform that corresponds to one of the spatial points is shown in Figure 2, representing only a small portion of the temporal information, for clarity. To filter the noise and improve the signal-to-noise ratio (SNR), temporal wave processing is performed in two stages. First, a bandpass filter is applied on all the waveforms. Second, mean value subtraction is performed, wherein the mean value of a specified wave is subtracted from all its sampling points.

Thus far, we have addressed the method for recording each ultrasonic wave separately and saving it as a series of data points in a dataset whose number is equal to the total number of points on which laser beam impinges. However, for the wavefield information to be visualized and the ultrasonic wavefield propagation images to be produced, we need to systematically sort them into groups.

**Figure 3:**
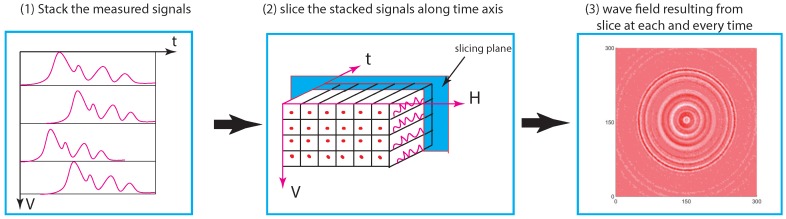
Scheme of an ultrasonic wave propagation imaging algorithm.

According to the UWPI scheme, sorting refers to the stacking of the measured signals on a vertical plane, i.e., the waveforms of the type shown in Figure 2 are arranged on a spreadsheet containing all the laser-beam-impinging points. That is, we construct a vertical plane comprising the impinging points as well as piles of planes in perpendicular directions comprising the time axis. This results in a 3D data structure. A horizontal plane slicing along the time axis at a specific temporal point can be applied to obtain a wave propagation image corresponding to that time. This is repeated for all the discrete time points, and a cluster of wavefield snapshots/images is produced by rendering the resulting 2D data. Finally, to produce an ultrasonic wave propagation movie, the snapshots are played in rapid succession in the order of their corresponding time points. The process layout in Figure 3 depicts the procedure of UWPI.

## 3. Formulation of Lamb Wave Frequency-Wavenumber Equation

A Lamb wave is a type of a propagating elastic perturbation within a solid plate [29]. Thus, it is in principle a guided wave existing between the upper and lower boundaries of a plate whose faces are traction-free. In contrast to bulk wave formulation, guided wave formulation requires the consideration of boundary conditions because it constitutes repeated interactions with boundaries as well as mode conversions of longitudinal waves and shear waves [30]. In addition, bulk waves are different from guided waves in that there are a limited number of modes (i.e., longitudinal and shear) in the former case, whereas an infinite number of modes exist in the latter case.

**Figure 4:**
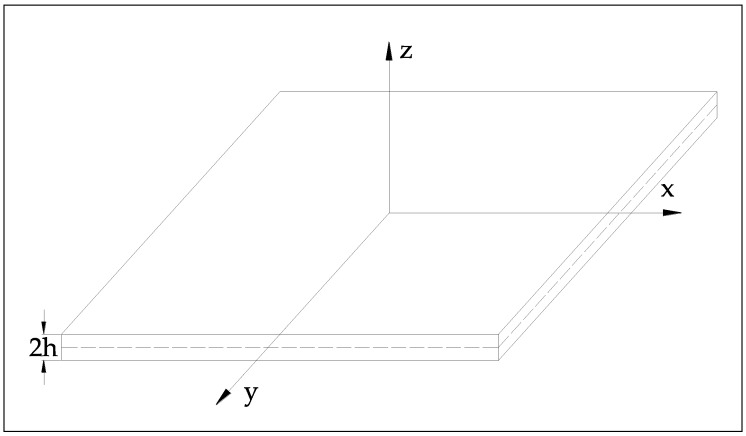
Geometry of a plate for formulating Lamb wave equation.

The Lamb wave dispersion relation is formulated based on the elastodynamic theory. Considering the model geometry in Figure 4, the particle displacement in a thin isotropic and homogenous plate can be stated as follows:

(1) $$\mu \;\cdot u_{i,ij}+\left(\lambda +\mu \right)\cdot u_{j,ji}+\rho \cdot f_{i}=\rho u_{i}^{\prime \prime },\left(i,j\right)=\left(x,y,z\right)$$

where μ and ρ are the shear modulus and density, respectively, of the plate. $u_{i}$ and $f_{i}$ represent the displacement and body force, respectively, in the *i* direction. λ is the Lam*è* constant and is expressed as $\;\lambda =\frac{2\mu .\nu }{1-2\nu }$. Here, ν is Poisson’s ratio.

For a finite medium, boundary conditions are imposed in terms of displacement and traction. Thenceforth, an exact solution is sought. There are two well-established approaches to deriving an exact solution: the displacement potential and partial wave techniques. Both methods were thoroughly discussed by Auld [31]. In addition, the former was discussed extensively by Achenbach [32]. Here, we discuss the displacement potential approach for completeness.

The concept of the potential method is to decompose the displacement field into vector and scalar potentials, φ and ψ, respectively. Evidently, it is feasible to express Equation (1) as follows, using vector notation:

(2) $$\mu \nabla^{2}u+\left(\lambda +\mu \right)\nabla \nabla \cdot u=\rho u^{\prime \prime }$$

The decomposition into vector and scalar potentials is of the following form:

(3) u=∇φ+∇×ψ

Substitute Equation (3) into (2) to obtain

(4) $$\mu \nabla^{2}\left[\nabla \varphi +\nabla \times \psi \right]+\left(\lambda +\mu \right)\nabla \nabla \cdot \left[\nabla \varphi +\nabla \times \psi \right]=\rho \frac{\partial^{2}}{\partial t^{2}}\left[\nabla \varphi +\nabla \times \psi \right]$$

It is known that $\nabla \cdot \nabla \varphi =\nabla^{2}\varphi$ and ∇·∇×ψ=0. Considering the two equations and rearranging terms in Equation (4), we obtain

(5) $$\nabla \left[\left(\lambda +2\mu \right)\nabla^{2}\varphi -\rho \varphi^{\prime \prime }\right]+\nabla \times \left[\mu \nabla^{2}\psi -\rho \psi^{\prime \prime }\right]=0$$

The non-trivial solutions to Equation (5) are then determined by equating the two terms in the equation to zero. Thus, we obtain two important relations that define the two types of bulk waves, i.e., longitudinal and shear waves. Equation (6) corresponds to longitudinal waves, whereas Equation (7) corresponds to shear waves.

(6) $$\nabla^{2}\varphi =\frac{1}{{c_{L}}^{2}}\varphi^{\prime \prime }$$

(7) $$\nabla^{2}\psi =\frac{1}{{c_{T}}^{2}}\psi^{\prime \prime }$$

where ${c_{L}}^{2}=\;\frac{\lambda +2\mu }{\rho }$ and ${c_{T}}^{2}=\;\frac{\mu }{\rho }$. For a plane strain problem in the x–y plane, the displacement in the y-direction is set to zero. Therefore, Equations (6) and (7) reduce to two dimensions and can be stated as follows:

(8) $$\frac{\partial^{2}\varphi }{\partial x^{2}}+\frac{\partial^{2}\varphi }{\partial z^{2}}=\frac{1}{{c_{L}}^{2}}\frac{\partial^{2}\varphi }{\partial t^{2}}$$

(9) $$\frac{\partial^{2}\psi }{\partial x^{2}}+\frac{\partial^{2}\psi }{\partial z^{2}}=\frac{1}{{c_{T}}^{2}}\frac{\partial^{2}\psi }{\partial t^{2}}$$

To proceed with the step-wise derivations for determining the expressions for the Lamb wave modes, an assumption is made with regard to the solutions of the two preceding relations, i.e., Equations (8) and (9). The assumptions are based primarily on the employment of expressions for the field variables representing a standing wave in the z-direction and a propagating wave in the x-direction [32].

(10) φ=Φ(z)exp[i(kx−ωt)]

(11) ψ=Ψ(z)exp[i(kx−ωt)]

Equations (10) and (11) are substituted into Equations (8) and (9), respectively, to determine Φ(z) and Ψ(z). The term i(kx−ωt) is omitted because it appears in all the expressions,

(12) $$\begin{array}{c} \Phi \left(z\right)=A_{1}sin\left(pz\right)+A_{2}cos\left(pz\right)\\ \Psi \left(z\right)=B_{1}sin\left(qz\right)+B_{2}cos\left(qz\right) \end{array}$$

where $p^{2}=\;\frac{\omega^{2}}{{c_{L}}^{2}}\;-\;k^{2}$ and $q^{2}=\;\frac{\omega^{2}}{{c_{T}}^{2}}\;-\;k^{2}$. Because the boundary conditions are imposed in terms of displacement and stress/traction, equations relating the displacement components with the potentials and those relating stress with the potentials are also considered. The relationships for the former case can be derived from Equation (3), whereas those for the latter are based on Hooke’s law. Because Φ(z) and Ψ(z) involve sine and cosine functions, the field variables (*u* and *w*, displacements in the x- and z-directions, respectively), which are expressed in terms of these two functions, also exhibit similar properties. Thus, the solution is split into two sets of modes: symmetric and antisymmetric modes. With these considerations and after performing the necessary rearrangements, we obtain

(13) $$\begin{array}{c} Symmetric\;modes:\\ \Phi \left(z\right)=A_{2}cos\left(pz\right)\\ \Psi \left(z\right)=B_{1}sin\left(qz\right)\\ u=ikA_{2}cos\left(pz\right)+qB_{1}cos\left(qz\right)\\ w=-pA_{2}sin\left(pz\right)-ikB_{1}sin\left(qz\right)\\ \tau_{zx}=\mu \left[-2ikpA_{2}sin\left(pz\right)+\left(k^{2}-q^{2}\right)B_{1}sin\left(qz\right)\right]\\ \tau_{zz}=-\lambda \left(k^{2}+p^{2}\right)A_{2}cos\left(pz\right)-2\mu \left[p^{2}A_{2}cos\left(pz\right)+ikqB_{1}cos\left(qz\right)\right] \end{array}$$

(14) $$\begin{array}{c} Antisymmetric\;modes:\\ \Phi \left(z\right)=A_{1}sin\left(pz\right)\\ \Psi \left(z\right)=B_{2}cos\left(qz\right)\\ u=ikA_{1}sin\left(pz\right)-qB_{2}sin\left(qz\right)\\ w=pA_{1}cos\left(pz\right)-ikB_{2}cos\left(qz\right)\\ \tau_{zx}=\mu \left[2ikpA_{1}cos\left(pz\right)+\left(k^{2}-q^{2}\right)B_{2}cos\left(qz\right)\right]\\ \tau_{zz}=-\lambda \left(k^{2}+p^{2}\right)A_{1}sin\left(pz\right)-2\mu \left[p^{2}A_{1}sin\left(pz\right)-ikqB_{2}sin\left(qz\right)\right] \end{array}$$

The boundary conditions are $\tau_{zx}=\tau_{zz}=0$ at z=±h. The application of these conditions yields two homogeneous equations for the two constants in each of the two modes (i.e., $A_{2}$ and $B_{1}$ for the symmetric modes and $A_{1}$ and $B_{2}$ for the antisymmetric modes). The determinant of these homogeneous equations must vanish for a non-trivial solution. After rearrangements, we obtain

(15) $$\frac{tan(qh)}{tan(ph)}=-\;\frac{4k^{2}pq}{{\left(q^{2}-k^{2}\right)}^{2}}\;\;\;\;\;\;\;for\;symmetric\;modes$$

(16) $$\frac{tan(qh)}{tan(ph)}=-\;\frac{{\left(q^{2}-k^{2}\right)}^{2}}{4k^{2}pq}\;\;\;\;\;\;\;for\;antisymmetric\;modes$$

## 4. Experimental Setup

In this study, an Nd:YAG pulsed laser was used to generate Lamb waves, and an AE (Acoustic Emission) sensor was used to measure the waveforms. The system components are shown in Figure 1. The principles of operation of the system are described here briefly. The laser is a Q-switched diode-pumped high-power solid-state Nd:YAG pulsed laser. The corresponding wavelength is 532 nm, whereas the maximum pulse repetition rate is 20 Hz. The pulse duration is 8 ns. The maximum energy per pulse of the laser is 55 mJ [33]. However, in this study, only a marginal fraction of the energy was used: 4% and 5% of the maximum laser energy were considered in this work. The criteria for deciding the margin of the energy is twofold. The first one is the requirement to keep the wave generation within the thermoelastic regime or to avoid any damage to the specimen. The second one, which is the criterion for deciding the lower limit of the laser energy utilized, is the fact that the intensities lower than 4% produced a low magnitude wavefield and thus are prone to alteration due to noise. The Q-switched Nd:YAG pulsed laser emits a laser beam through the galvanometer after the trigger signal is transmitted to it [34]. The mirrors in the galvanometer are then tilted immediately to direct the laser beam at the target point along the scanning path. The sensor measures the signal and transmits the measured value to the data digitizer. Then, the digitized signal reaches the image processor, where the UWPI process occurs.

**Figure 5:**
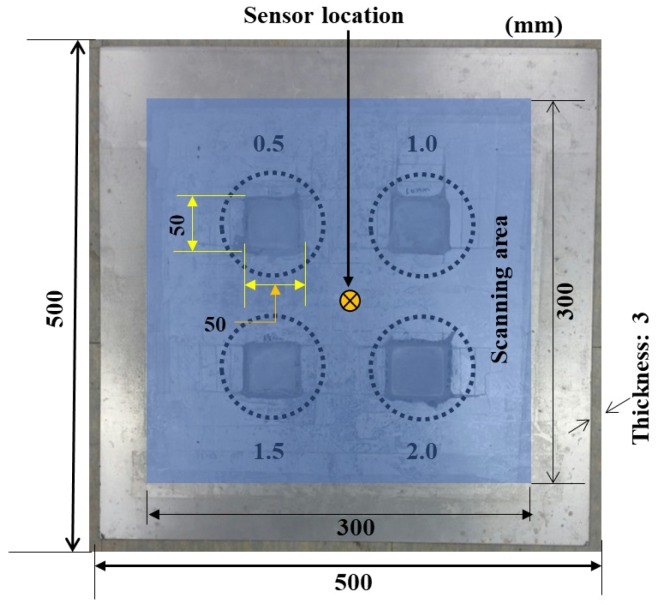
Layout of specimen: corroded regions and scanning area.

The specimen used in this study is a 6061-T6 aluminum plate of size 500 mm × 500 mm and thickness 3 mm. The physical and mechanical properties of the 6061-T6 aluminum are listed in Table 1. Four corrosion regions were artificially developed on a face of the sample. These were developed using concentrated hydrochloric acid. As the primary objective of this study was to investigate the feasibility of determining the corrosion depth, the depth of the corroded area was made to vary. The depth values used are 0.5, 1.0, 1.5, and 2.0 mm. In contrast, the area of each of the four regions was 50 mm × 50 mm. The specimen is shown in Figure 5. The specimen was fixed on a metal frame by tightly clamping the bottom part of the specimen with two clamps to the supporting frame.

Laser scanning was performed on an area of 300 × 300 mm that enclosed all four of the corroded regions. The sensing component (here, a broadband AE sensor) was situated at the center of the scanning area. The scanning process was carried out in the vertical direction starting from the bottom-right end of the scanning area. Each time that the top or the bottom margin was reached, the scanner moved to the left and scanned the adjoining line of points in a similar fashion. This is illustrated in Figure 1. The interval between the points in both the vertical and horizontal maneuvers was set to 2 mm. Therefore, we have 151 × 151 = 22,801 points in total.

The mechanism of wave generation was in the thermoelastic regime, to prevent damage to the sample. A waveform was generated and propagated at each laser impinging point as the scanner moves. Then, the sensor recorded the wave amplitude information with respect to time. The sensor measurement was such that the registration of wave information corresponding to a specified point was completed before the waveform generated at the immediate next point arrived. Therefore, at the completion of the scanning operation, there were totally 22,801 separate wave forms.

## 5. Results and Discussion

### 5.1. UWPI and Damage Visualization

The output of the UWPI illustrates the interaction of the wavefield with the flaws on the specimen under inspection. We successfully visualized the interaction of the wave with each of the corroded regions by applying the UWPI technique. Furthermore, feature sensitive parameters such as RMS could be computed from the data compiled during the UWPI process.

(17) $$w_{RMS}\left(x,y\right)\_W^{k}\;=\sqrt{\frac{1}{T}\sum_{t=1}^{T}{\left(w\left(x,y,t\right)\right)}^{2}*t^{k}}$$

where *T* is the total measurement time of the signals, k is the weighting parameter (here, equal to two), and w(x,y,t) is the wave signal. $w_{RMS}\left(x,y\right)\_W^{k}\;$ is a weighted RMS function. The weight was applied because the magnitude of the propagating wave decreases with distance from the excitation; thus, larger RMS values are exhibited near the excitation [35]. This makes it challenging to identify damages farther from the excitation. Therefore, the weight equalized the RMS in the whole area. The RMS could locate the damages more clearly than the ultrasonic wave propagation movie. The RMS distribution provided the required information on the coordinates of each side of the corroded areas.

**Figure 6:**
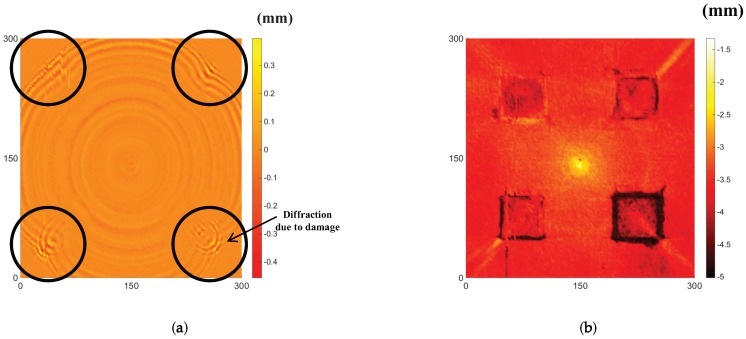
Results of applying UWPI: (**a**) snapshot of a propagating wave; and (**b**) plot of the cumulative RMS values.

**Figure 7:**
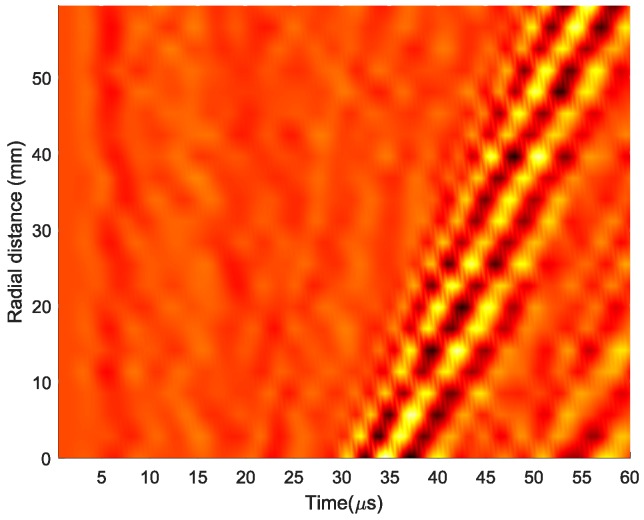
Position–time–amplitude map for a wave propagating in a corroded region.

Once the coordinates of the corroded areas were determined, we could employ UWPI and obtain the wave information within each area. As evident from the snapshot of UWPI in Figure 6a, the wave propagated radially outward from the center of the plate (i.e., position of the sensor). This is because of the reciprocity theorem, which states that, for a linear system, the wave responses measured at a point from a wave generated at all the scanning points is equivalent to the response at each scanning point if the wave had been generated at the receiver [36]. Therefore, a straight line passing through each corroded region can be drawn starting from the center. The position–time–amplitude values of the wave on the portion of the straight line where it traversed the corroded region under consideration were extracted. Figure 7 illustrates a position–time–amplitude map corresponding to the wave propagation in the corroded region of depth 0.5 mm. The y-axis represents the radial distance from the nearest point to the sensor along the line drawn. The position–time–amplitude map is converted to a wavenumber–frequency–amplitude map via a Fourier transformation.

### 5.2. Proposed Method: Corrosion Depth Determination

**Figure 8:**
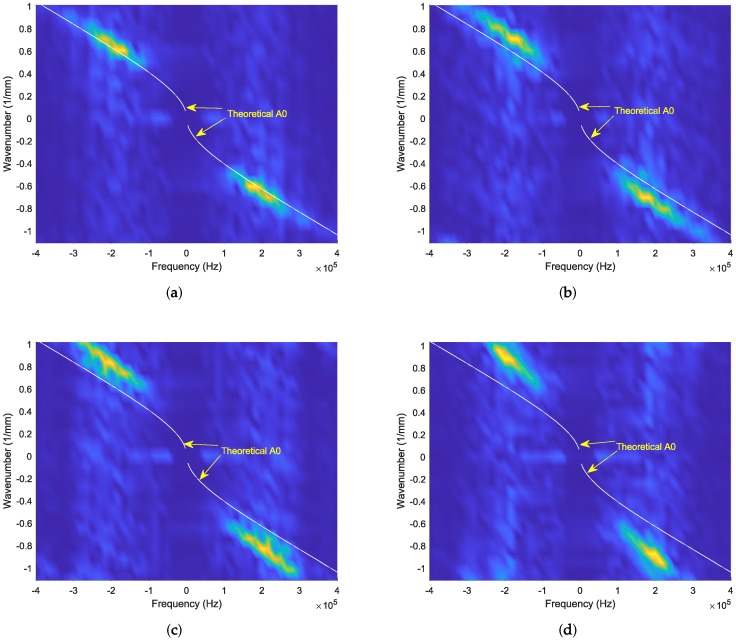
Dispersion curves of the wave propagating through: (**a**) 0.5mm deep corroded region; (**b**) 1mm deep corroded region; (**c**) 1.5mm deep corroded region; and (**d**) 2mm deep corroded region.

A dispersion curve for a real Lamb wave on the specimen can be computed and plotted following the procedure described in the next sentences. Because wave propagation is required in the corroded area under consideration, it is necessary to localize the analysis to that area. The experimental dispersion curve corresponding to the wave in the corrosion-damaged region can be determined from the Fourier transformed position–time–amplitude values described in the previous section. Figure 8a–d illustrates the dispersion curves. In these figures, it is apparent that only one Lamb wave mode is measurable within range. This is the high-energy Lamb wave mode with the lowest cut-off frequency, which appears on the graph, i.e., the $A_{0}$ mode. The theoretical dispersion curve of the $A_{0}$ mode is also drawn alongside the experimental ones. The theoretical dispersion curve is drawn using the properties of 6061-T6 aluminum listed in Table 1 and an undamaged plate thickness of 3 mm. It is evident from the results in Figure 8a–d that the theoretical curves are offset from the experimental ones. Moreover, the level of offset differs across the regions. The lower the is damage depth, the smaller is the deviation. Therefore, the graph that represents corrosion damage with a depth of 0.5 mm should be the closest to the theoretical curve. In contrast, the graph that represents a corrosion damage depth of 2 mm should be the greatest deviation from the theoretical curve. Therefore, in general, as the damage severity increases, the theoretical and experimental curves move farther away from each other. Figure 8 shows this to be the case. Therefore, damage severity can be predicted from these types of plots.

However, in this study, the aim was to quantify the depth of the damage. Thus, depth determination was performed using the information obtained from the graphs. Hereafter “the analysis” refers only to the antisymmetric Lamb wave mode for the reason mentioned in the previous sentences. Equation (16) represents the spectrum of the antisymmetric Lamb wave mode. It contains the parameter frequency, wavenumber, and plate thickness. Frequency–wavenumber pairs can be identified from plots of the kind shown in Figure 7 by tracing the path of the experimental curve. In contrast to the theoretical curve, the experimental plot does not exhibit a one-ω-to-one-*k* relationship. It is a ridge-type plot containing more than one ω for a specified *k*. Owing to the one-*k*-to-many-ω characteristics, the depth computation was repeated a number of times, and a weighted average was computed. The weighting parameter is the magnitude of the wave at the ω–*k* pair under consideration. The objective was to compute for the thickness using Equation (16). The equation can be re-expressed as follows:

(18) $$tan(qh)+\;\frac{{\left(q^{2}-k^{2}\right)}^{2}}{4k^{2}pq}\;tan(ph)=0$$

For a known $c_{L}$, $c_{T}$, and ω–*k* pair, the only unknown in Equation (18) is the parameter h, which denotes half the thickness of the plate. $c_{L}$ and $c_{T}$ were computed to be 6149 and 3097 m/s, respectively. Equation (18) was computed numerically for h in MATLAB${}^{®}$ using the function vpasolve. An initial estimate or search range was introduced into the function as half the undamaged plate thickness, which is 1.5 mm. This was to reduce the computation effort and cost. The results of the computation were inserted into Equation (19) to obtain the weighted thickness. Its outcomes are presented in Table 2. The corrosion depths could then be obtained by subtracting the weighted thickness from the undamaged plate thickness (i.e., 3 mm). These are presented in Table 2.

(19) $$weighted\;average\;thickness=\frac{\sum {h_{i}Z}_{i}}{\sum Z_{i}}$$

where $h_{i}$ are the half-thickness values computed using the extracted ω–*k* pairs. $Z_{i}$ are the corresponding wave magnitudes.

The pulsed laser scanning process can be varied by varying the laser intensity setting. The variation occurs in terms of the energy of the laser beam. A fraction of the maximum energy of the Nd:YAG pulsed laser used in this study (i.e., 55 mJ) was used to generate an ultrasonic wave thermoelastically. In this regard, the results in Table 2 correspond to the scanning process performed using 4% of the maximum laser energy.

Table 2 reveals that the technique determined the depths of all the four damaged areas fairly accurately. The predicted value of the lowest corrosion damage depth was also approximately equal to the nominal depth. Therefore, the technique can quantify sub-millimeter corrosion depths. It is noteworthy that the capability of the method to determine a sub-millimeter damage depth is highly important in that it enables the solution of the problem, i.e., corrosion damage, at an early stage. In addition, because the primary concern of an NDE is to detect and quantify minute flaws and because it is feasible to recognize a large-sized defect with the naked eye, this result can be considered as an achievement that has a high potential for various applications. The higher-size damages are also quantified accurately, which demonstrates the robustness of the method.

As mentioned in the preceding paragraphs, the energy of the input laser beam can be varied. The variation in the energy in turn causes a difference in the ultrasonic wave generated and propagated on the target plate. Therefore, it would be desirable to examine the validity of the results for a different laser intensity, although the present results are adequate. In this view, the experimental procedure outlined in the previous section was followed, and the specimen was scanned again by altering the laser intensity from 4% to 5%. This setting was altered using the PC, on which a LABVIEW${}^{®}$-based system synchronization setting and a corresponding GUI was setup. Thereafter, all the procedures required for determining the corrosion depths were repeated similarly to the case with 4% laser intensity. The resulting values are presented in Table 3. The dispersion plots of the type presented in Figure 8 can also be displayed. These new plots are highly similar to those obtained in the earlier case and therefore, thus are not presented here. Again, in Table 3, all four depths are mostly accurate. In addition, the values in the figure are almost equal to the corresponding values in the case with 4% laser intensity. The accuracy of determination of sub-millimeter corrosion depths is, again, maintained. On the other hand, the smallest defect size which the method is capable of determining its depth should be investigated. We examined the smallest possible corroded area our method can determine its depth. This was accomplished by progressively reducing the spatial distance between the end points considered for the position–time–amplitude map and by performing the depth determination each time. As a result, the smallest defect area whose depth was reliably determined was 6 mm × 6 mm.

## 6. Conclusions

This study addressed the detection, localization, and size determination of corrosion damage on an aluminum plate. Damage detection and localization were carried out using the UWPI technique of wave visualization followed by flaw imaging with the aid of the computation of the RMS of the wavefield. Because wavefield visualization and flaw imaging provide aerial information regarding the damage and do not enable the quantification of the depth of the damage, a method is proposed for accomplishing this task. As the target specimen is a plate, the analysis should be performed by assessing the properties of Lamb waves propagating within the specimen, particularly within the corroded areas. To achieve this, the theoretical Lamb wave equation (the Rayleigh–Lamb equation) was used to predict the depths of the damages. Prior to this, the damage-centered position–time–amplitude values of the wave were transformed to the corresponding wavenumber–frequency–amplitude values via a Fourier transform.

The theoretical dispersion curves were drawn alongside the experimental dispersion plots. The differences between the plots drawn for the four damaged areas indicate that they represent different depths. The differences in terms of the proximities of the experimentally determined curves to the theoretical dispersion curves were considered because the latter represent the intact plate of thickness 3 mm, whereas the former represent the damaged areas, which have lesser thicknesses. Accordingly, the plot corresponding to the smallest damage depth is the most similar to the theoretical plot, and the one corresponding to the largest depth is the farthest.

Apart from the comparison of the damage depths based on the closeness of the theoretical dispersion plots to the experimental ones, accurate size determination was also performed. This was performed by extracting frequency–wavenumber pairs from the experimental dispersion plots and inserting them into the Rayleigh–Lamb equation. The only unknown here was the thickness. Only the mode with a high energy and the lowest lower cut-off frequency (antisymmetric mode A${}_{0}$) was observed in the experimental dispersion curve. Therefore, the computation was based only on this mode. It is apparent that a similar result would be obtained when other modes are also present on the plots and are used for the computation. The experimental magnitude map exhibits a ridge-type appearance, indicating the existence of multiple wavenumbers for a specified frequency, or vice versa, in contrast to the theoretical curves. That is, if a theoretical dispersion curve is plotted for the thickness corresponding to each damaged region, the magnitude map would appear to be distributed on top and to its left and right. Therefore, the depth was computed for multiple frequency–wavenumber pairs, and a weighted average was taken. Two laser intensities were used while the specimen was scanned. Therefore, the analysis was repeated twice. The results for both the laser intensity values were highly close to being accurate, revealing the robustness of the proposed method.

## Figures and Tables

**Table 1:** Properties of the 6061-T6 aluminum plate employed in this study.

Density (g/cc)	Young’s Modulus (GPa)	Shear Modulus(GPa)	Poisson’s Ratio
2.7	68.9	26	0.33

**Table 2:** Results of numerical computation of the thickness of a corrosion-damaged region on the plate (laser intensity = 4.0%).

Artificial Corrosion Depth	Weighted Average Thickness (2h)	Predicted Corrosion Depth
0.5 mm	2.442 mm	0.558 mm
1.0 mm	1.864 mm	1.136 mm
1.5 mm	1.555 mm	1.445 mm
2.0 mm	1.159 mm	1.841 mm

**Table 3:** Results of numerical computation of the thickness of a corrosion-damaged region on the plate (laser intensity = 5.0%).

Artificial Corrosion Depth	Weighted Average Thickness (2h)	Predicted Corrosion Depth
0.5 mm	2.470 mm	0.530 mm
1.0 mm	1.827 mm	1.173 mm
1.5 mm	1.530 mm	1.470 mm
2.0 mm	1.151 mm	1.849 mm

## Abbreviations

The following abbreviations are used in this manuscript:

Nd:YAG	Neodymium-doped Yttrium Aluminum Garnet
NDE	Non-Destructive Evaluation
PZT	Lead Zirconate Titanate
UWPI	Ultrasonic Wave Propagation Imaging
AE	Acoustic Emission
RMS	Root Mean Square
SLDV	Scanning Laser Doppler Vibrometer
GUI	Graphic User Interface
SNR	Signal-to-Noise Ratio
